# Brucellosis in the Addis Ababa dairy cattle: the myths and the realities

**DOI:** 10.1186/s12917-018-1709-4

**Published:** 2018-12-14

**Authors:** Bedaso Mammo Edao, Gizachew Hailegebreal, Stefan Berg, Aboma Zewude, Yemiserach Zeleke, Teshale Sori, Gizat Almaw, Adrian M. Whatmore, Gobena Ameni, James L. N. Wood

**Affiliations:** 1 Disease Dynamics Unit, Department of Veterinary Medicine, University of Cambridge, Madingley Road, Cambridge, CB3 0ES UK; 2 College of Veterinary Medicine, Addis Ababa University, P.O.Box 34, Bishoftu, Ethiopia; 3 South Agricultural Research Institute, Worabe Agricultural Research Center, P.O.Box 21, Worabe, Ethiopia; 4 Animal and Plant Health Agency, Woodham Lane, Addlestone, KT15 3NB UK; 5 National Animal Health Diagnostic and Investigation Centre, Sebeta, Ethiopia; 6 Aklilu Lemma Institute of Pathobiology, Addis Ababa University, P.O.Box 1176, Addis Ababa, Ethiopia

**Keywords:** Addis Ababa, Brucella, Dairy cattle, Ethiopia, Risk factors, Seroprevalence

## Abstract

**Background:**

Bovine brucellosis is considered as an important disease among livestock and people in sub-Saharan African countries including Ethiopia. A cross-sectional study was conducted from November 2016 to May 2017 to estimate the prevalence and associated risk factors, and to assess knowledge-attitude and practices (KAP) of farm workers about bovine brucellosis in Addis Ababa dairy farms.

**Results:**

A total of 1550 cattle from 127 dairy farms were serially tested using the Rose Bengal Plate Test (RBPT), Competitive Enzyme-Linked Immunosorbant Assay (c-ELISA) and Complement Fixation Test (CFT). Forty-three (2.77%) of the collected sera were positive by the RBPT and only one of these was positive by c-ELISA (0.06%) and none was positive by CFT. The knowledge of farm workers towards the disease was very low and risk factors associated with *Brucella* infection were apparent in the study area.

**Conclusion:**

Seropositivity for *Brucella* spp. was found in only a very small percentage by c-ELISA test, although risk factors for transmitting *Brucella* infection were present. The results suggest that bovine brucellosis is currently not a generalized problem in dairy cattle of Addis Ababa. Since this favorable disease situation is not the result of informed policy, there is no guarantee that it will continue unchanged. Setting clear policy in control of the disease and implementing “One Health” are the most constructive approaches recommended.

**Electronic supplementary material:**

The online version of this article (10.1186/s12917-018-1709-4) contains supplementary material, which is available to authorized users.

## Background

Ethiopia has one of the largest cattle populations in Africa [1] despite gaining minimum return from this resource as a result of various technical and non-technical factors, including infectious diseases. Bovine brucellosis is one of the infectious diseases hampering productivity of cattle and has been reported from several parts of the country [2] Bovine brucellosis is a zoonotic disease with economic and public health impact, particularly for human and animal populations in developing countries that rely mainly on livestock production [3]. The disease can generally cause significant loss of productivity through abortion, stillbirth, low herd fertility and low milk production [4].

Bovine brucellosis is considered to be predominantly caused by *Brucella abortus*; and, to a much lesser extent by *B. melitensis*, where cattle are kept together with infected goat or sheep. It is characterized clinically by abortion at first gestation, retained fetal membrane (RFM), metritis, orchitis and epididymitis [5, 6]. Sources of infection for the transmission of the bovine brucellosis are aborted fetuses, retained fetal membranes, and vaginal discharges and milk from infected animals. Direct contact with an aborting cow and the aborted fetus and indirect contact with contaminated fomites are the most common means of transmission of the disease in cattle. Ingestion of contaminated feed, fodder, water and grazing on contaminated pasture, may also play a secondary role in disease transmission [5, 7]. The disease has been eradicated from many developed countries; it however, remains a major public and animal health problem in many developing countries, where rural income relies mainly on livestock and dairy products [8]. Brucellosis in human often originates from domestic animal reservoir and associated with various risk factors and behavioral practices such as consumption of raw milk and milk products and close contact with infected animals [9, 10].

The risk factors that influence the spread and maintenance of brucellosis are related to management systems, the genetic content of susceptible animal population, biology of agents causing the disease and environmental factors. These include the size and composition of the herd, age of the animals, frequent contact between infected and susceptible herds, poor farm biosecurity and climate change [5, 11].

In Ethiopia there is no documented information on how and when bovine brucellosis was introduced and established. However, in the last two decades several serological surveys have showed that it is endemic and widespread [2, 12–26]. These studies in animals and humans were largely confined to serological surveys and commonly targeted cattle, occasionally sheep and goats, and rarely camels. So far, there was only one attempt to identify *Brucella* species in the country [27]; the distribution and proportion of their natural hosts was also not studied exhaustively [19]. According to [28], *Brucella* seroprevalence in dairy cattle of Ethiopia revealed highest prevalence in central Ethiopia followed by southern part whereas lowest prevalence was revealed in western part of the country.

Market oriented new dairy industries are emerging in Ethiopia so as to contribute hugely towards filling the gap between an increasing national demand and supply for milk and milk products. Cow dairy development roadmap of the country is aimed at increasing the productivity of indigenous cattle through improvements in genetics, health and feeding to satisfy consumption demand and start export of cow milk and milk products. It is important that public and private dairy industries and cooperatives require up to date and consistent scientific data on bovine brucellosis. This would in turn assist in developing baseline information to establish nationwide bovine brucellosis intervention policies aimed at controlling and eradicating the disease.

Thus, this study was designed to estimate the seroprevalence of brucellosis in dairy cattle, to identify potential risk factors and assess knowledge, attitude and practices (KAP) of the farm workers towards the disease.

## Results

### Knowledge-attitude and practices (KAP) of the farm workers about brucellosis

#### Demographic characteristics

Out of 127 surveyed farms, 130 farm workers in 59 farms were willing to participate in the KAP study. Of the 130 farm workers responsible for the management of the farm, the majority (88%) was male, and more than 50% were between ages 25 to 60 years. Half of the participants were not married (51.5%) and most of them had family size below 10 people. Eighty percent of the respondents had attended only primary school and more than 5 % were illiterate (Table 1).

**Table 1:** Demographic characteristics of farm workers in the study area (*n* = 130)

Demographic characteristics	Category	number	Percent
Gender	Female	16	12.3
	Male	114	87.7
Age	12–24	54	41.5
	25–60	72	55.4
	> 60	4	3.1
Marital status	Single	67	51.5
	Married	63	48.5
Level of education	Illiterate	9	6.9
	Primary	104	80
	Secondary	13	10
	Technical/Diploma	3	2.3
	Degree	1	0.8
No of people in the household^a^	1–5	61	46.9
	6–10	67	51.5
	> 10	2	1.6

^a^Household defined as family members regularly living together and sharing meals, *n* number

#### Risk factors

Most of the study participants reported several risk factors for acquiring bovine brucellosis. The majority of participants, 96% of farm workers in small scale, 100% in medium size and 92.6% in large herd sized farms were not aware of bovine brucellosis. Most respondents, 83.6% in small scale, 60% in medium size and 81.5% in large farms disposed of dead fetus/after birth to open dump in the environment and more than 5 % of participants in small herd sized farms fed aborted materials to dogs. Almost all participants in small scale and medium sized farms and 77.8% in large-scale farms practiced assisted parturition without wearing protective gloves or masks. More than 60 and 80% percent of farm workers in all farm sizes consume raw milk and meat, respectively (Table 2).

**Table 2:** Knowledge-attitudes and practices of farm workers about *Brucella* infection in the study area

Variables	Proportion of respondents (n)		
	Herd size		
	Small (*n* = 73) n (%)	Medium (*n* = 30) n (%)	Large (*n* = 27) n (%)
Awareness about brucellosis			
Yes	3 (4)	0 (0)	2 (7.4)
No	70 (96)	30(100)	25 (92.6)
Dead fetus/After birth disposal			
Burning/Burying	7 (9.6)	12 (40)	5 (18.5)
Open dump	61 (83.6)	18 (60)	22 (81.5)
Give to dog	5 (6.8)	0 (0)	0 (0)
Assist parturition			
Yes	72 (98.6)	30(100)	21 (77.8)
No	1 (1.4)	0 (0)	6 (22.2)
Consume raw milk			
Yes	50 (68.5)	18 (60)	20 (74)
No	23 (31.5)	12 (40)	7 (26)
Consume raw meat			
Yes	60 (82.2)	25(83.3)	24 (88.9)
No	13 (17.8)	5 (16.7)	3 (11.1.)

*n* number

### Farm characteristics

Of the 127 farms, 103, 17 and 7 were small, medium and large herd sized farms, respectively. Of the farms assessed by a questionnaire survey, more than 70% of medium and large farms, as well as 42.7% of small sized farms, had reproductive problems (abortion, stillbirth, retained fetal membrane and repeat breeding) in their farms. The majority of farms were using artificial insemination (AI) for breeding purposes and 71.4% of large sized farms raised their own replacement animals whereas most of small and medium sized farms used both (raised their own stock and purchase). The practices of provision of separate pens for parturition and aborted animals were 28.6 and 14.3% in large sized farms, respectively, whereas there were no such practice in small and medium sized farms and almost all farms used flushing with tap water to clean pens after parturition. The majority (85.7%) of the large herd sized farms, and more than 40% of medium and small-scale farms, used separate feed and water supply for each animal. Reproductive problems and age were prominent culling criteria in all farms and the majority of farms (> 70%) in the study area did not report frequent contact of dairy animals with other species (sheep and goat) (Table 3).

**Table 3:** Summary of characteristics of dairy farms in the study area

Variables	Herd size		
	Small (*n* = 103) Frequency (%)	Medium (*n* = 17) Frequency (%)	Large (n = 7) Frequency (%)
Reproductive problems			
Yes	44 (42.7)	12 (70.6)	5 (71.4)
No	59 (57.3)	5 (29.4)	2 (28.6)
Service type			
AI	71 (69)	9 (53)	5 (71.4)
Bull Both	9 (8.7) 23 (22.3)	4 (23.5) 4 (23.5)	0 (0) 2 (28.6)
Replacement stock			
Raised own	31 (30.1)	8 (47)	5 (71.4)
Purchased Both	5 (4.9) 67 (65)	1 (6) 8 (47)	0 (0) 2 (28.6)
Parturition pen			
Yes	0 (0)	0 (0)	2 (28.6)
No	103 (100)	17 (100)	5 (71.4)
Cleaning pen after parturition			
Flushed with tap water	101 (97)	17 (100)	7 (100)
Flushed with water and disinfect	2 (3)	0(0)	0 (0)
Separate pen for aborted cow			
Yes	1 (1)	0 (0)	1 (14.3)
No	102 (99)	17 (100)	6 (85.7)
Feed and water supply			
Own	50 (48.5)	7 (41.2)	6 (85.7)
Communal	5 (4.9)	4 (23.5)	0 (0)
Both	48 (46.6)	6 (35.3)	1 (14.3)
Culling criteria			
Reproductive problem	57 (55.3)	6 (35.3)	4 (57.1)
Logistics	7 (6.8)	3 (17.7)	0 (0)
Age	39 (37.9)	8 (47)	3 (42.9)
Contact with other spp. ^a^			
Yes	12 (11.6)	4 (23.5)	2 (28.6)
No	91 (88.4)	13 (76.5)	5 (71.4)

Contact with other spp. ^a^ Sheep and goat

### Seroprevalence of bovine brucellosis in dairy cattle

A total of 1550 dairy cattle were tested with RBT and 43 (2.77%) of them were positive in this test. The RBT positive sera samples were further tested using c-ELISA and CFT. Only one animal was confirmed seropositive for bovine brucellosis in the study area based on c-ELISA and no sero reactor animal was found by CFT. In addition to RBT positive sera samples, equal number of randomly selected RBT negative sera were shipped to APHA, Weybridge, UK and further tested using RBT and C-ELISA. However, the result was the same. The seroprevalence of bovine brucellosis in Addis Ababa dairy farms was thus 0.06% (1/1550) based on c-ELISA test. The herd level seroprevalence of brucellosis based on c-ELISA was 0.8% (1/127). The prevalence of antibodies to *Brucella* spp. in small, medium and large sized cattle farms was 0, 5.8% (1/17) and 0%, respectively (Table 4).

**Table 4:** Individual animal level and herd level prevalence of bovine brucellosis in dairy farms of Addis Ababa

Farm type	Individual farm			Herd level				
	No tested	RBT Positive n^a^ (%)	C-ELISA Positive n^a^ (%)	CFT Positive n^a^ (%)	No Tested	RBT Positive n^a^ (%)	C-ELISA Positive n^a^ (%)	CFT Positive n^a^ (%)
Small	821	5(0.6)	0(0)	0(0)	103	5(4.8)	0(0)	0(0)
Medium	363	9(2.5)	1(0.27)	0(0)	17	6(35.3)	1(5.8)	0(0)
Large	366	29(7.9)	0(0)	0(0)	7	3(42.9)	0(0)	0(0)
Total	1550	43(2.77)	1(0.06)	0(0)	127	14(11)	1(0.8)	0(0)

*n*^a^ number positive

## Discussion

Improvement of knowledge-attitudes and practices among urban dairy farm workers could have a significant impact on the reduction of many zoonotic infections, including brucellosis. The results of the KAP study show that the majority of farm workers in the studied dairy farms were not aware of bovine brucellosis (96.1%). Farm workers with a primary and lower level of education were less likely to have heard of brucellosis when compared to those with secondary and higher level of education. Animal attendants with a primary and lower level of education are hence likely at a higher risk of exposure to the disease. Similar findings were reported by a study conducted in Tajikistan [29]. Low awareness of the disease in the study area might be explained by the low prevalence of brucellosis in dairy cows. The majority (88%) of farm workers were male. This could be due to the fact that farm works in urban and peri-urban intensive diary is labor demanding, as a result of which most farm owners prefer to employ male farm workers.

Practices posing a high risk of *Brucella* transmission are very common: most participants reported assisting in animal parturition, disposing aborted fetuses/after birth in open environment without protective gloves or masks and consumption of raw meat and milk. The reason could be poor knowledge of the disease and risks of transmission but also lack of resources used for personal protection such as gloves, aprons and antiseptics. Creation of awareness of the farm owners and attendants is important to control brucellosis in the area even though the prevalence in animals was low in this serological survey.

In the present study, bovine brucellosis at individual animal level was 0.06% (1/1550) and herd level prevalence was 0.8% (1/127) using c-ELISA whereas no seropositive animal was found on CFT in dairy farms of Addis Ababa. This observation is consistent with previous reports made by [30] in Eastern and Western Showa zones of central Ethiopia using Rose Bengal Plate test (RBPT), serum agglutination test (SAT) and complement fixation test (CFT) (*n* = 564). This report is also consistent with [31], who reported no positive reactors in intensive dairy farms of the Addis Ababa area (*n* = 747). Similarly, a study by [23] could not find positive reactors in Adama, central Ethiopia (*n* = 52) and northern Ethiopia (Mekele and Gondar) (*n* = 252). A study conducted in Debrebirhan and Ambo towns by [32] reported that there was only one sero-reactor animal to *Brucella* infection using CFT(*n* = 415).

In contrast, there are reports of a higher prevalence of antibodies to *Brucella* spp. in Addis Ababa dairy farms, 1.5% [11] and 2.2% [23]. A similar study by [26] reported 1.4% in Asella and Bishoftu towns using the card test (CT), RBPT, indirect Enzyme-Linked Immuno Sorbant Assay (i-ELISA) and Complement Fixation Test (CFT). In Ethiopia, brucellosis in animals has been reported from different localities of the country; with prevalence ranging from 0.4 to 8% particularly associated with cattle in both intensive and extensive management systems [2, 11–15, 18–25]. A high seroprevalence of brucellosis (38%) in cattle in western Ethiopia has been reported [33], while most of the studies suggested a low seroprevalence (below 5%) in cattle.

In the present study only one sero reactor animal to *Brucella* spp. was found in cattle populations of Addis Ababa dairy farms. According to the individual animal record in the farm, this seroreactor animal in the farm was purchased from outside the capital and had late abortion history at first calving. This is remarkable, as bovine brucellosis is considered the world’s most common bacterial zoonosis [34] and listed among top five zoonotic diseases in Ethiopia [35]. The hypothesis that brucellosis is endemic in the investigated dairy farms of Addis Ababa could thereby not be confirmed in the present study. However, the presence of one or more positive reactors in the herd is a reliable predictor of the presence of infection [5]. Seropositivity for *Brucella* spp. was found in only a very small percentage by c-ELISA test, although risk factors for obtaining *Brucella* infection such as, age variety, origin of animals, different level of parity, history of abortion, and herd size composition were present as revealed by farm characteristics analysis.

In epidemiological studies, the use of two or more tests applied serially is recommended to maximize the accuracy of test results. RBPT is highly sensitive test and c-ELISA and CFT are highly specific and also sensitive and usually used as confirmatory tests [36]. The combination of these tests in this study could therefore maximize the accuracy of the findings. False positive serological reactors in RBT are due to cross-reactions with Smooth Lipopolysaccharide (S-LPS) antigens of other bacteria. As there has never been history of vaccination, seropositivity in this case is due to natural infection.

The difference in test results of C-ELISA and CFT is due to the variation in sensitivity of the tests. The C-ELISA test is more sensitive as compared to CFT for the diagnosis of brucellosis even though both tests have similar specificities of 99.9% [36]. Moreover, CFT is prone to prozone effect (low dilutions of some titrated sera from infected animals do not fix complement) that could lead to a false negative result [37, 38].

The very low prevalence could be explained by the cross sectional study design if informal culling practices suggested by [17] had been instituted. These include culling of cows with abortion history of at least two times and above for any reason and removing seropositive reactors from the herd for economic reasons. The other possible explanation might be absence of infectious foci, such as *Brucella*-infected dairy farms or ranches in the surrounding areas, which could spread the disease among contact herds. Movement of infected animals to susceptible herds is a common route of transmission [5]. The random selection used in our study design should have detected clustered infection if it were common [39].

## Conclusions

Urban and peri-urban dairy farming offers an important opportunity to improve the livelihood of people in low-income countries. There was only one seropositive animal found for bovine brucellosis in the present study by the c-ELISA test. This study showed poor knowledge of brucellosis and abundant high-risk behaviors among the farm workers. Poor knowledge and high-risk practices strengthens the logic for including health education as part of control programs. At present, there is no officially coordinated program for control of bovine brucellosis in Ethiopia. The disease is unlikely to be a significant limiter of dairy production in the Addis Ababa area due to the low prevalence, but it may be present in other animal types. Since this favorable disease situation is not the result of informed policy, there is no guarantee that it will continue unchanged.

The current study warrants the need for constant surveillance program in case the prevalence rates do change. Implementing a “*test and slaughter*” program in the zero grazing system to eliminate the existing low risk of brucellosis could avert the cost-related limitation of brucellosis control. Modernization of husbandry systems and testing of new animals before introducing to dairy farms should be encouraged. A multi-sectorial framework should be promoted involving all stakeholders working in public and animal health in the context of a “One Health” approach. Since the current study was limited to cross sectional study design, future studies in the dairy farms should follow longitudinal study types to ascertain actual burden of the disease in the study area.

## Methods

### Description of the study area

**Fig. 1:**
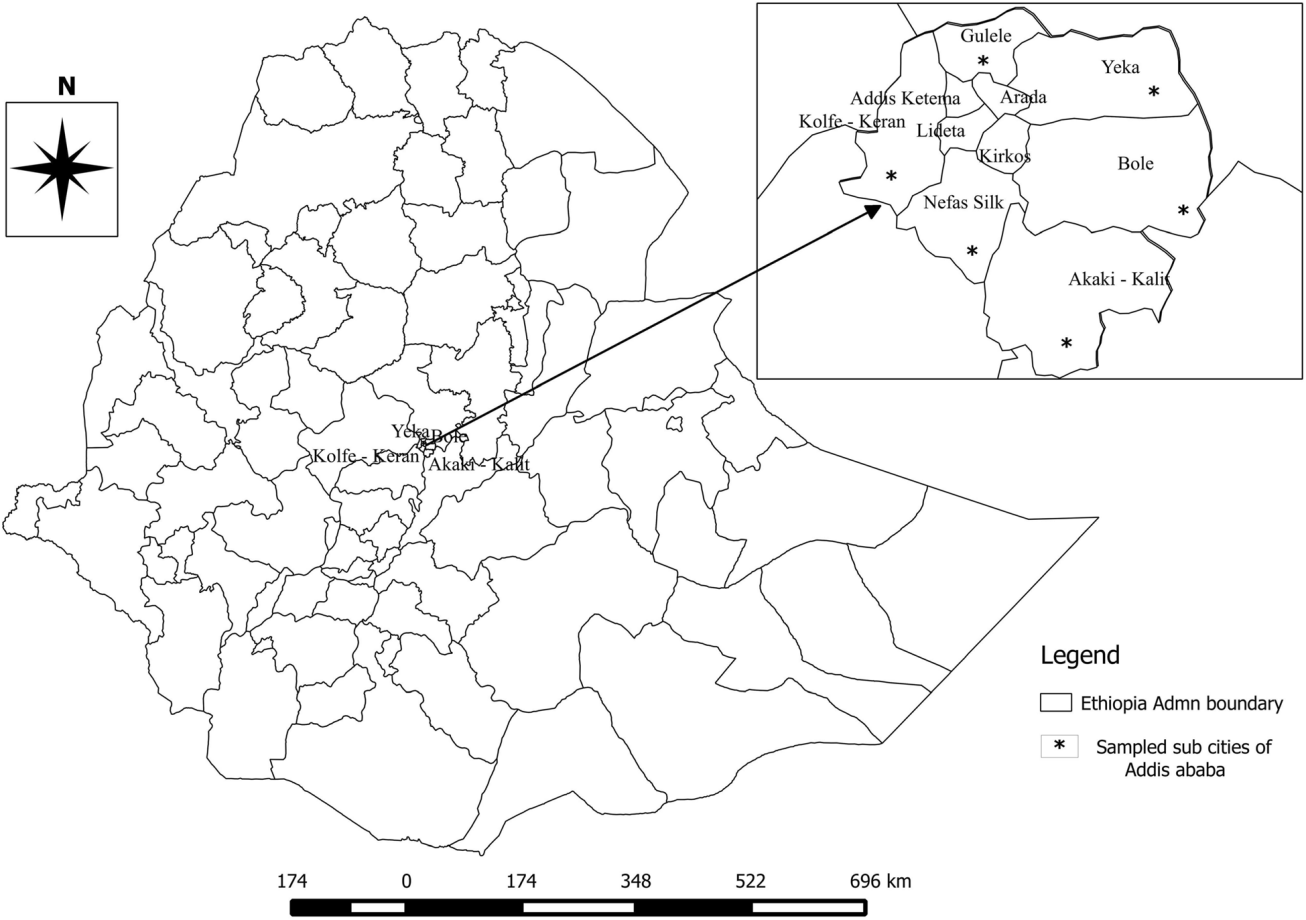
Map of Addis Ababa showing studied/sampled sub cities. The map depicted in Fig. 1 is our own developed from Ethiopian shape files using QGIS software

The study was conducted in Addis Ababa dairy farms. Addis Ababa, the capital city of Ethiopia, lies at an elevation of 2300 m (7,500 ft) above sea level and is featured by a grassland biome. It is geographically located at 9°1′48″N latitude and 38°44′24″E longitude. It has a typical highland climate with temperature ranging from 11 °C - 24 °C. Addis Ababa has a mean annual rainfall of 1300 mm with bimodal distribution. The city is divided into 10 boroughs (Fig. 1), called sub cities, and 99 wards (kebeles) [40].

Dairy cattle production systems in Ethiopia are classified into commercial dairy systems, urban and peri-urban smallholder dairy, rural smallholder (mixed crop and livestock production), and pastoral and agro pastoral [23]. The dairy systems hold mainly exotic breeds or crosses with the local zebu breeds, while the rural husbandry systems stock mainly zebus. This study focused on urban and peri-urban smallholder farms, which produce milk for home use and sale, and commercial dairy systems, which are producing milk as a full-time business. These dairies constitute the main dairy source for the capital and produce milk for sale [26]. Within these systems dairy farms were classified based on size of the herd and herd management into large scale farms, with more than 50 animals, medium scale farms (20 to 50 animals) and smallholder farms (< 20 animals).

### Variables collected

Explanatory variables that were hypothesized to be risk factors for the disease were assessed at both individual animal and farm level. Information related to herd structure was extracted from individual herd records and when this was not the case from farm owners or managers interview using a pre tested structured questionnaires. Herd level parameters studied include herd size as described above, the presence of reproductive problems such as abortion, retained fetal membranes and still birth in the farm, separate calving pen, brucellosis testing in the farm, frequent contact with animals in other herds and species, which were categorized as yes or no variables. The major reasons for culling were coded either as reproductive problems, old age or logistics. Breeding strategy was artificial insemination, bull or both. The afterbirth (aborted fetuses and fetal membranes) disposal method was also categorized into burying, open dump or feeding to dogs. The feeding and water supply strategies were classified into communal and own.

Individual animals were categorized as young 6–17 months or adult (≥17 months), breed as Holstein-Friesian (HF), cross, or local. The origin of each individual animal was defined as either own stock or purchased. The clinical reproductive disorder experience, such as abortion, stillbirth and retained fetal membrane were categorized as either yes or no variables. Parity number and frequency of some of the aforementioned disorders were also recorded. Physiological status of an animal was categorized as pregnant, non-pregnant, lactating and lactating-pregnant. Repeat breeding was also assessed based on the animal owner’s general observation. Accordingly, cows that demanded 3 or more services per pregnancy were categorized as a repeat breeder otherwise they were categorized as a non-repeat breeder.

### Study population

The target study populations were dairy cattle above six months of age, which consist of breeding females, replacement heifers, and available bulls. The breeds of these animals were crosses of local breed with HF. None of the animals tested were vaccinated against brucellosis. For the Knowledge, Attitude and Practice (KAP) study, occupationally associated farm workers, willing to be interviewed, were included.

### Study design and sample size

A cross-sectional study design was employed from November 2016 to May 2017 to address the objectives of the study. According to Addis Ababa city urban agriculture bureau there are about 880 registered smallholder, medium scale and large commercial dairy farms in Addis Ababa. Individual farm was considered as a herd and the primary sampling unit. The sample size for dairy farms was calculated using a 9.1% herd level seroprevalence of bovine brucellosis [23], 95% confidence interval (CI) and 5% required precision [41]. Hence, a total of 127 dairy farms were considered for this study and proportional allocation was made for each sub city based on the number of farms. In each sub city, herd-sampling frame was established in collaboration with sub city veterinary department and farms were selected randomly using computer generated random numbers. Before data collection, consent was made with the identified farms owners requesting their farms to be included in the study. Farms where the owners were not willing to participate in the study were replaced by other farms. All cattle above six months of age in the selected dairy farms were included and a total of 1550 animals were sampled for serological screening. For the KAP study farm workers from sero surveyed farms, who agreed to be interviewed, were included. Hence, 130 farm workers from 59 farms participated in the study.

### Data collection

Data concerning farm workers KAPs towards the disease were collected by interviewing individuals using a pre-tested structured questionnaire. Verbal consent was obtained from the respondents and the objective of the survey explained to them before start of the interview. The interviews were conducted in local languages (Afaan Oromo or Amharic). The questionnaire focused on demographic characteristic of the interviewee, knowledge-attitude about the disease, handling and afterbirth/aborted fetus disposal practices, habit of raw animal product consumption and animal feeding and housing practices.

Blood samples (10 ml) from the jugular vein of each animal were collected, using sterile needles and plain vacutainer tubes labelled with individual animal identification number. The blood samples were centrifuged at 3000×g for 10 min to obtain the serum within 12 h of collection. Sera were decanted into cryo-vials, identified and stored at − 20 °C until screened for antibodies against natural *Brucella* exposure using serological analysis.

### Rose Bengal plate test (RBPT)

All sera samples collected were initially screened by RBPT using RBPT antigen (Animal and Plant Health Agency, New Haw, Addlestone, Surrey, KT15 3NB, United Kingdom) according to OIE (2016) procedures. Sera and antigen were taken from refrigerator and left at room temperature for half an hour before the test to reach room temperature. Briefly, RBT antigen (30 μl) was added onto a glass slide next to an equal amount of cattle sera. The antigen and test serum were mixed thoroughly in a plastic applicator, shaken for 4 min, and agglutination was read immediately. Any observed agglutination by the naked eye was considered to be a positive reaction.

### Competitive ELISA

All RBPT positive sera were further tested using the COMPELISA 160 and 400, a competitive ELISA kit for the detection of antibodies against *Brucella* in serum samples (Animal and Plant Health Agency, New Haw, Addlestone, Surrey, KT15 3NB, United Kingdom) at Addis Ababa University, Aklilu Lemma Institute of Pathobiology (AAU-ALIPB). The test was performed according to the manufacturer’s instructions. The test was conducted in 96-well polystyrene plates that are pre-coated with *Brucella* species lipopolysaccharide (LPS) antigen. 20 μl of each test serum was added to each well followed by 100 μl of prepared conjugate solution. The plates were then shaken vigorously for two minutes and incubated at room temperature for 30 min on rotary shaker, at 160 revs/min. Plates were washed 5 times and dried. Hydrogen peroxidase substrate and chromogen solution was developed for 10 min. 100 μl of o-Phenylenediamine dihydrochloride (OPD) solution was added to all wells and the plates were incubated at room temperature for 10 to 20 min. Micro plate reader was switched on and the units allowed stabilizing for 10 min. The reaction was then being stopped using stopping solution. Optical densities (OD) were read at 450 nm using micro plate reader. The lack of color development indicated that the sample tested was positive. A positive/negative cut-off was calculated as 60% of the mean of the OD of the 4 conjugate control wells. Any test sample giving an OD equal to or below this value was regarded as being positive. An animal was considered positive if it tested seropositive on both RBPT and c-ELISA in serial interpretation.

### Complement fixation test (CFT)

All samples that were RBPT-positive were further subjected to complement fixation test as a confirmatory test at the National Veterinary Institute (NVI), Bishoftu, Ethiopia. The *Brucella* antigen and control sera (positive and negative) used during the test were produced by Animal and Plant Health Agency, UK. The standardization of the antigen was made at 1:20 working dilution (strength). The *Brucella* antigen, complement and 3% sensitized sheep red blood cells were added after the test sera were serially diluted (1:5, 1:10, 1:20, and 1:40) in microtitre plates. Then the plates were incubated at 37 °C for 30 min. The test was considered positive when the reading was as partial fixation (50% haemolysis) or complete fixation (no haemolysis) at 1:10 dilution. The validity of the test was considered when there was complete hemolysis in negative control serum and the positive control shows inhibition of hemolysis.

### Case definition

Animals were considered positive to brucellosis when they tested positive on either RBPT/CFT or RBPT/c-ELISA tests in parallel interpretation. Similarly, a herd or farm was considered seropositive when at least one animal in a herd or farm tested positive. Since there is no history of vaccination against brucellosis in Ethiopia, seropositivity observed in this study was considered to be due to natural infection.

### Data analysis

Data generated from the questionnaire survey and laboratory investigations were recorded and coded using a Microsoft Excel spreadsheet (Microsoft Corporation) and analyzed using STATA version 13.0 for Windows (Stata Corp. College Station, TX, USA). Descriptive statistics were used for demographic and farm characteristics as well as KAPs relating to bovine brucellosis. The seroprevalence was calculated as the number of seropositive samples divided by the total number of samples tested.

## Additional files

Additional file 1: Raw Data (XLSX 136 kb)

Additional file 2: Questionnaire prepared to assess the knowledge, attitude and practices (KAP) of farm workers towards brucellosis in Addis Ababa, Central Ethiopia. (DOCX 22 kb)

## Abbreviations

AAU-ALIPB: Addis Ababa University, Aklilu Lemma Institute of Pathobiology
AW: Adrian Whatmore
AZ: Aboma Zewde
BM: Bedaso Mammo
cELISA: Competitive Enzyme Linked Immunosorbent Assay
CFT: Complement Fixation Test
CI: Confidence Interval
GA: Gobena Ameni
GH: Gizachew Hailegebreal
GZA: Gizat Almaw
HF: Holstein Friesian
iELISA: Indirect Enzyme Linked Immunosorbent Assay
JW: James Wood
KAP: Knowledge Attitude Practice
LPS: Lipopolysaccharide
NVI: National Veterinary Institute
RBPT: Rosbengal Plate Test
RFM: Retained Fetal Membranes
SAT: Serum Agglutination Tests
SB: Stefan Berg
TS: Teshale Sori
YZ: Yemiserach Zeleke
ZELS: Zoonoses and Emerging Livestock Systems
