# Supplementary material for: Brucellosis in the Addis Ababa dairy cattle: the myths and the realities
Source: BMC Vet Res. 2018 Dec 14;14:396. doi: 10.1186/s12917-018-1709-4 (PMC6293529; doi:10.1186/s12917-018-1709-4)
Supplement: Supplementary file 2 — Questionnaire prepared to assess the knowledge, attitude and practices (KAP) of farm workers towards brucellosis in Addis Ababa, Central Ethiopia. (DOCX 22 kb) [file 12917_2018_1709_MOESM2_ESM.docx]

**Questionnaire prepared to assess the knowledge, attitude and practices (KAP) of farm workers towards brucellosis in Addis Ababa, Central Ethiopia.**

Dear participant,

This survey is an investigation of risk factors precipitating the transmission of bovine brucellosis to humans and assessment of knowledge practices and attitudes of farm workers toward bovine brucellosis. The results of this study will help veterinarians and public institutions in designing control strategies.

There is no need to tell your name to the enumerator. Your responses will be kept confidential. You will in no way be personally linked to any of the results of the survey. There is no risk to you from participating in this questionnaire, and there is no anticipated direct benefit. Thank you in advance for your participation!

**Part I: Demographic issues**

1. Sex: Male ☐Female ☐
2. Age: Below 13 ☐, 13-19 ☐ 20-59 ☐ above 60 ☐
3. Residence: Urban ☐ Periurban ☐ Rural ☐
4. Marital status: Married ☐ Single ☐ Widowed ☐ Divorced ☐
5. Animals at home: Yes ☐ No ☐
6. What the last grade of formal education you completed?

No formal school ☐ Some primary ☐ Completed primary ☐ Some secondary school ☐ Completed secondary school ☐ Technical /vocational ☐ Some/completed pre-university ☐ Completed diploma degree ☐University ☐ Don’t know ☐ Refused ☐

1. How many people live in your house hold (including children, relatives)?
2. Other comments? ……

**Part II: Awareness of Brucellosis**

1. Have you heard of the disease brucellosis? Yes ☐ No ☐ If yes, from where did you get the information?

Veterinarians ☐ public health workers ☐ newspapers ☐ TV ☐

1. Which animal can get infected with brucellosis?…………
2. Can humans be infected with brucellosis? Yes ☐ No ☐ If yes, what symptoms ……….
3. Do you know how spread occurs between animals? Yes ☐No ☐
4. Do you know brucellosis as a zoonotic disease? Yes ☐No ☐
5. Does brucellosis present like any other illnesses? Yes ☐No ☐
6. If “yes” to the above question, which other illnesses look like brucellosis? A) Malaria b) typhoid c) tuberculosis d) others e) I don’t know
7. Do you know how humans can be infected with brucellosis from an animal? Do not read the options

Insect bites ☐

By close contact with infected animals☐

by consumption of raw milk/milk products ☐

by consumption of raw meat ☐

handling aborted foetuses and placentas, offals ☐

assisting during animal during calving/abortion ☐

contact with infected people☐

others ☐, please specify …………

Don’t know ☐

1. Do you know if there is any treatment for brucellosis in cows/sheep/goats? Yes ☐ No ☐ If yes, what kind and for how long?
2. Is brucellosis treatable in human? Yes ☐No ☐
3. Who do you talk to most regularly about animal health issues?

Family member/friend ☐

neighbour ☐

Veterinarian ☐

Village chief /community leader ☐

Other, please specify ………….

**Part III: Attitudes**

Skip question 1,2 and 3 if the answer was NO on the question “ have you heard of the disease Brucellosis”(Part I:1)

1. Do you believe any family members are at risk of acquiring brucellosis? Yes ☐ NO ☐
2. If yes, to the above questions, which family member(s) do you think is /are most susceptible to infection?..............
3. If any animal in your household gets brucellosis, how serious do you consider this to be?

Cattle: Not serious ☐ quite serious ☐ Very serious ☐

Sheep: Not serious ☐ quite serious ☐ Very serious`

Goats: Not serious ☐ quite serious ☐ Very serious

Do you need/would you like more information on brucellosis? Yes ☐ No ☐ If yes, how would you like to receive that information?.........................

**Part IV: Practices**

Skip question 6 if the answer was NO on the question “Do you involve in delivery of pregnant cow?”

1. How often do you milk cows per day?
2. How often do you wash your hands after milking the cows? Every time ☐Frequently ☐ sometimes ☐Rarely ☐Never ☐
3. If answering to above question some times, rarely, never –why?

Not important ☐ No soap/not enough soap ☐ No clean water ☐ other reason ☐ specify

1. What do you do with dead foetuses (Calf, lamb, kid)……………
2. Do you take any specific actions to protect yourself when dealing with cows having an abortion or with retained placenta/dead foetuses? Don’t read out.

Use gloves ☐ use mask ☐ wash hands ☐ others ☐please specify..

1. Do you involve in delivery of pregnant cow? Yes: ☐ NO: ☐
2. If yes to the question number 5 above, how do you involve?

Birth aid ☐ Assistance to veterinarians ☐ Giving IU medication ☐

1. Do you involve in routine intra uterine medication? Yes ☐ No ☐
2. Do you drink fresh milk? Yes ☐ No ☐ If no, what you do with the raw milk/milk products before consumption…………..
3. Do you consume raw meat? Yes ☐ No ☐ If no, what you do with the raw meat before consumption…………..
